# The first seroepidemiological survey for *Angiostrongylus vasorum* in domestic dogs from Romania

**DOI:** 10.1186/s13071-019-3481-0

**Published:** 2019-05-14

**Authors:** Georgiana Deak, Nina Gillis-Germitsch, Angela Monica Ionică, Angela Mara, Ioana Raluca Păstrav, Cristina Daniela Cazan, Mariana Ioniță, Ioan Liviu Mitrea, Cristian Răileanu, Diana Bărburaș, Maria Nedișan, Răzvan Oachiș, Vasile Cozma, Roland Schaper, Manuela Schnyder, Andrei Daniel Mihalca

**Affiliations:** 1 Department of Parasitology and Parasitic Diseases, University of Agricultural Sciences and Veterinary Medicine Cluj-Napoca, Cluj-Napoca, Romania; 2 Institute of Parasitology, Vetsuisse-Faculty, University of Zurich, Zurich, Switzerland; 3 Department of Parasitology and Parasitic Diseases & Animal Biology, Faculty of Veterinary Medicine, University of Agronomic Sciences and Veterinary Medicine of Bucharest, Bucharest, Romania; 4 Department of Public Health, Faculty of Veterinary Medicine, University of Agricultural Sciences and Veterinary Medicine, Iaşi, Romania; 5 Federal Research Institute for Animal Health, Institute of Infectology, Friedrich-Loeffler-Institute, Riems, Germany; 6 SC Veterra SRL, Sighişoara, Romania; 7 Bayer Animal Health GmbH, 51368 Leverkusen, Germany

**Keywords:** Canine angiostrongylosis, Romania, *Angiostrongylus vasorum*, Serology, Antigen and antibody detection

## Abstract

**Background:**

*Angiostrongylus vasorum* is a metastrongyloid nematode localized in the right heart and the pulmonary arteries of domestic dogs. The number of reports in Europe has recently increased, presumably as a consequence of a growing awareness among clinicians, animal owners and researchers, but also due to a growing incidence and territorial spread. So far, no studies have been conducted to assess the prevalence and distribution of *A. vasorum* in domestic dogs in Romania, and the awareness among veterinarians is limited or absent. The aim of the present study was to evaluate the countrywide seroprevalence of circulating antigens of *A. vasorum* and specific antibodies against *A. vasorum* in domestic dogs from Romania.

**Methods:**

Between November 2016 and July 2017, blood was sampled from a total of 1545 domestic dogs from 23 counties of Romania. Details about their gender, age, breed, housing, use and origin were collected. All serum samples were tested for the presence of *A. vasorum* circulating antigens (AG) using monoclonal and polyclonal antibodies in a sandwich ELISA. Additionally, a sandwich ELISA using *A. vasorum* adult somatic antigen purified by monoclonal antibodies was used for specific antibody (AB) detection.

**Results:**

A total of 33 dogs (2.14%, 95% CI: 1.82–3.56%) were seropositive for *A. vasorum* antigen or antibodies against the parasite. Three dogs were positive for antigen only (0.19%, 95% CI: 0.07–0.57%) and 30 dogs (1.94%, 95% CI: 1.36–2.76%) were positive for antibodies only. No dog was positive for both tests. The overall prevalence (AB or AG) and the AB prevalence were significantly higher in pure breed dogs compared to mixed breeds and mongrel dogs (*P *< 0.05) and in shepherd dogs compared to other groups (*P *< 0.05). There was no significant difference between males and females, between urban and rural dogs, between dogs with unrestricted access and with restricted access to the environment, and between dogs living outdoors and indoors.

**Conclusions:**

Our data suggest that the disease is present in Romania in dogs, as it was previously demonstrated in foxes. However, so far, no clinical case has been reported in the country and this may be related to a low awareness among vets.

**Electronic supplementary material:**

The online version of this article (10.1186/s13071-019-3481-0) contains supplementary material, which is available to authorized users.

## Background

*Angiostrongylus vasorum* is a metastrongyloid nematode localized in the right heart and the pulmonary arteries of mainly domestic dogs [1] and foxes (*Vulpes vulpes*) [2], but also in other wild carnivores: gray wolves (*Canis lupus*) [3], golden jackals (*Canis aureus*) [4], coyotes (*Canis latrans*) [5], red pandas (*Ailurus fulgens*) [6], meerkats (*Suricata suricatta*) [7] and various mustelids, using mainly gastropods as intermediate hosts [8, 9] and possibly amphibians and birds as paratenic hosts [10]. Infection of definitive hosts may also occur by ingestion of vegetation, food or water contaminated with secretions from infected gastropods [1, 11].

Since its first description in 1853 in France [12], *A. vasorum* is presently considered to have a wide distribution and lately the number of reports in Europe has increased presumably as a consequence of a growing awareness among clinicians, owners and researchers, but also due to a growing incidence and territorial spread [13].

*Angiostrongylus vasorum* causes a wide range of clinical manifestations in dogs, the most frequent being respiratory signs (cough, dyspnoea), bleeding disorders (haemorrhages) and neurological symptoms, which may lead to severe or fatal outcomes [13–15]. This variety, in addition to further unspecific clinical signs, can make the diagnosis of canine angiostrongylosis challenging. A specific diagnosis can be reached using the reference standard technique (Baermann method), based on the detection of first-stage larvae (L1) in faecal samples [16]. The morphological identification of L1 requires expertise, as the larvae can be misidentified as that of other lungworms such as *Crenosoma vulpis* or *Filaroides* spp., as well as a variety of free-living nematodes that can contaminate the samples. FLOTAC represents another coproscopic method used for the detection of *A. vasorum* L1 in faecal samples, with a good sensitivity [17]. However, both coproscopic techniques have the disadvantage that they cannot detect prepatent infections. The prepatent period is 38–57 days, when damage to the lung parenchyma is already present [18, 19]. More recently, specific PCRs [20, 21] and serological methods [22, 23] have been developed for the identification of infected animals. Serological methods (ELISAs) are used for clinical diagnosis of individual cases but also for epidemiological screening [24]. Furthermore, a rapid commercial blood test is available for the detection of *A. vasorum* antigens in domestic dogs (IDEXX Angio Detect™, IDEXX Laboratories, Westbrook, ME, USA) with a sensitivity of 84.6% in clinically suspect dogs [25].

In Romania, the current occurrence of *A. vasorum* in domestic and wild canids is poorly known. The parasite was identified by necropsy in 4.2% of the red foxes from the western part of the country [26]. Larval stages resembling *A. vasorum* have been reported in faeces from dogs from the Timiș County, but they were not molecularly confirmed and the origin and travel history of the dogs was not specified [27]. So far, no studies have been conducted to assess the prevalence and distribution of *A. vasorum* in domestic dogs in Romania and the awareness among vets is limited or absent (Mihalca, personal communication).

The aim of the present study was to evaluate the countrywide seroprevalence of specific circulating antigens of *A. vasorum* and specific antibodies against *A. vasorum* in domestic dogs from Romania.

## Methods

Between November 2016 and July 2017, a total of 1545 domestic dogs from 23 counties of Romania were included in the study. Details about their gender, age, breed, housing, use and origin were collected (full data and categories used for statistical analysis are given in Additional file 1) for each dog. Details about the previous anthelmintic treatments were also collected, but due to the largely incomplete dataset on this point (no information on the date of the last treatment, no information on the product used, etc.) this was excluded from the data analysis. Blood samples (5–9 ml) were collected from the cephalic vein using S-Monovette 9 ml, Clotting Activator/Serum (Sarstedt, Nümbrecht, Germany). The serum was separated by centrifugation and stored at − 20 °C until use. All samples were tested at the Institute of Parasitology, Vetsuisse Faculty, University of Zurich, Switzerland, for the presence of *A. vasorum* circulating antigens (AG) using monoclonal and polyclonal antibodies in a sandwich ELISA, with a sensitivity of 95.7% and a specificity of 94.0%, as described by Schnyder et al. [22]. Additionally, a sandwich ELISA (sensitivity 81.0%, specificity 98.8%) using *A. vasorum* adult somatic antigen purified by monoclonal antibodies (mAb Av 5/5) was used for specific antibody (AB) detection [23]. Test thresholds were regionally determined based on the mean value of optical density (A_405_ nm) plus three standard deviations of 300 randomly selected samples [28]. All test runs included a background control, a conjugate control, three positive control sera from three experimentally infected dogs and two negative control sera from uninfected dogs.

The collected data were analysed using EpiInfo™ 7 software (CDC, Atlanta, GA, USA). The prevalence of AB and/or AG presence and corresponding 95% confidence intervals (95% CI) were calculated. Differences among the various categories were assessed by means of Chi-square testing and were considered statistically significant for values with *P *< 0.05.

## Results

**Fig. 1:**
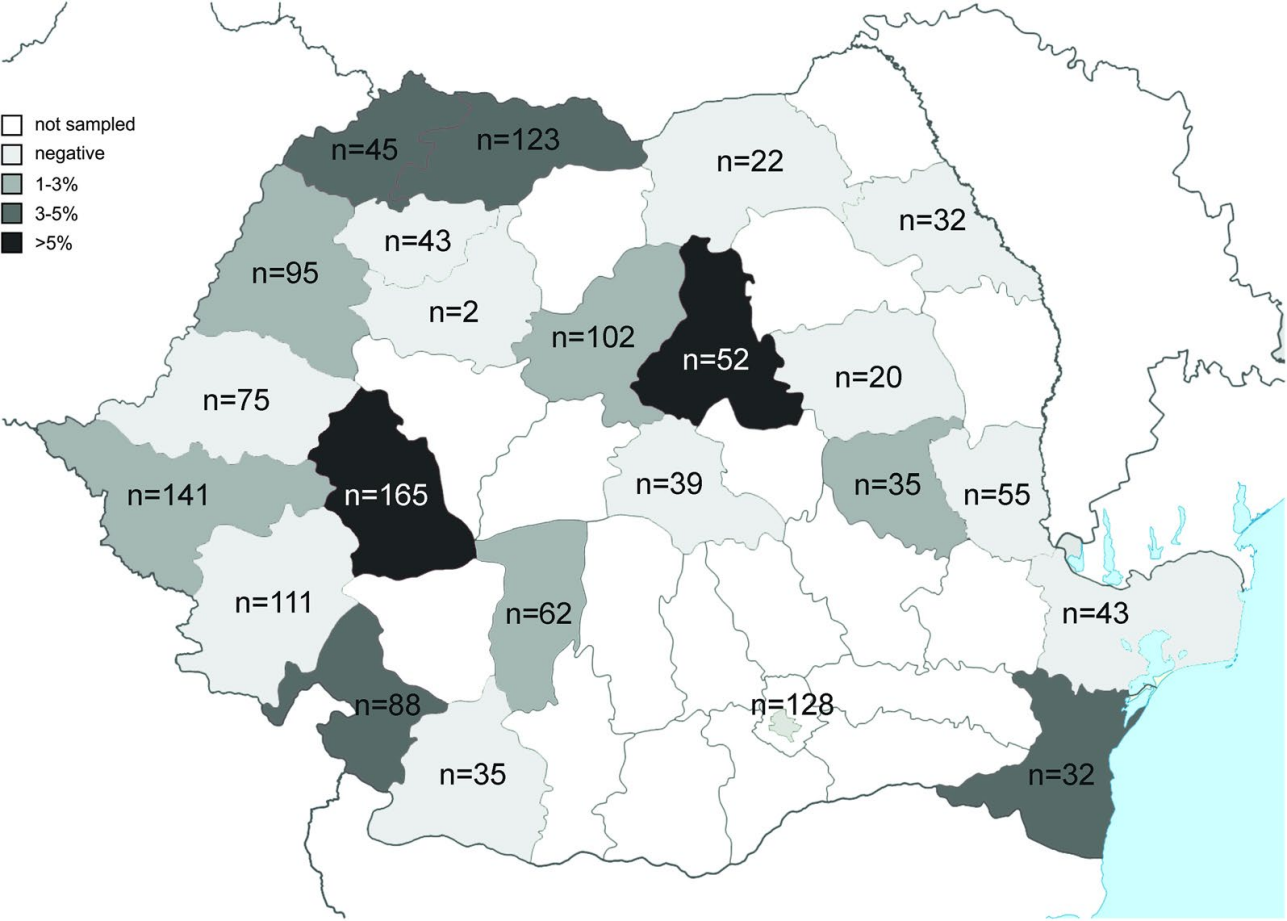
Seroprevalence of *A. vasorum* circulating antigens or antibodies by county. The number of sampled dogs is indicated for each county

A total of 33 dogs (2.14%, 95% CI: 1.82–3.56%) were seropositive for *A. vasorum* antigen or antibodies against the parasite. Three dogs were positive for antigen only (0.19%, 95% CI: 0.07–0.57%) and 30 dogs (1.94%, 95% CI: 1.36–2.76%) were positive for antibodies only. No dog was positive for both tests. The prevalence by county (AG or AB) varied between 1.61 and 6.06% (Table 1, Fig. 1). The prevalence for each dog category and statistical data are shown in Additional file 2.

**Table 1:** Seroprevalence of *A. vasorum* circulating antigens and antibodies by county in dogs from Romania (n=1545)

County	Examined	AG		AB		AG or AB	
		%	95% CI	%	95% CI	%	95% CI
Hunedoara	165	0	0–2.21	6.06	2.94–10.86	6.06	2.94–10.86
Harghita	52	0	0–6.85	5.77	1.21–15.95	5.77	1.21–15.95
Satu Mare	45	0	0–7.87	4.44	0.54–15.15	4.44	0.54–15.15
Maramureș	123	0.81	0.02–4.45	3.25	0.89–8.12	4.07	1.33–9.23
Mehedinți	88	1.14	0.03–6.17	2.27	0.28–7.97	3.41	0.71–9.64
Constanța	32	0	0–10.89	3.13	0.08–16.22	3.13	0.08–16.22
Vrancea	35	2.86	0.07–14.92	0	0–10.00	2.86	0.07–14.92
Timiș	141	0	0–2.58	2.13	0.44–6.09	2.13	0.44–6.09
Bihor	95	0	0–3.81	2.11	0.26–7.40	2.11	0.26–7.40
Mureș	102	0	0–3.55	1.96	0.24–6.90	1.96	0.24–6.90
Vâlcea	62	0	0–5.78	1.61	0.04–8.66	1.61	0.04–8.66
Arad	75	0	0–4.80	0	0–4.80	0	0–4.80
Bacău	20	0	0–16.84	0	0–16.84	0	0–16.84
Brașov	39	0	0–9.03	0	0–9.03	0	0–9.03
București	128	0	0–2.84	0	0–2.84	0	0–2.84
Caraș-Severin	111	0	0–3.27	0	0–3.27	0	0–3.27
Cluj	2	0	0–84.19	0	0–84.19	0	0–84.19
Dolj	35	0	0–10.00	0	0–10.00	0	0–10.00
Galați	55	0	0–6.85	0	0–6.85	0	0–6.85
Iași	32	0	0–10.89	0	0–10.89	0	0–10.89
Sălaj	43	0	0–8.22	0	0–8.22	0	0–8.22
Suceava	22	0	0–15.44	0	0–15.44	0	0–15.44
Tulcea	43	0	0–8.22	0	0–8.22	0	0–8.22
Total	1545	0.19	0.07–0.57	1.94	1.36–2.76	2.14	1.82–3.56

The overall prevalence (AB or AG) and the AB prevalence were significantly higher in pure breed dogs compared to mixed breeds and mongrel dogs (*χ*^2 ^= 6.264, *df*= 2, *P*= 0.043 for AB and *χ*^2 ^= 6.677, *df*= 2, *P*= 0.035 for AB or AG). Among service groups (use), the overall prevalence (AB or AG) and the AB prevalence were significantly higher in shepherd dogs compared to other groups (*χ*^2 ^= 10.463, *df*= 4, *P*= 0.033 for AB and *χ*^2 ^= 10.401, *df*= 4, *P*= 0.034 for AB or AG). When considering only the AB prevalence, there was a significant difference between counties (*χ*^2 ^= 34.32, *df*= 22, *P*= 0.045). However, when considering the overall prevalence (AB or AG), there was no significant difference between counties. Moreover, there was no significant difference between males and females, between urban and rural dogs, between dogs with unrestricted access and with restricted access to the environment, and between dogs living outdoors and indoors. The statistical analyses indicated a significantly higher overall (AB or AG) prevalence and AB prevalence in dogs aged below 6 months (*χ*^2 ^= 11.537, *df*= 3, *P*= 0.009 for AB and *χ*^2 ^= 11.043, *df*= 3, *P*= 0.011 for AB or AG).

## Discussion

Similar studies using the same diagnostic approach were performed in several countries across Europe (Table 2). The main difference so far is that Romania is the only country (except Bulgaria, where the sample size was small) where no dog was positive for both AG and AB detection. Generally, the percentage of such dogs was very low (0.28% in Italy to 1.36% in Hungary). Similarly to the case of Romania, most of the positive dogs included in the previous studies were positive only for the antibodies (0.56% in Italy to 3.11% in Slovakia). According to Schnyder et al. [25, 28, 29], the seropositivity only to antibodies may indicate a parasite exposure, meaning that the sampling took place (i) during the prepatency (i.e. between 3 and 5 weeks post-infection) when circulating antigens are not yet detectable (detection starts between 5 and 11 weeks post-infection); (ii) after death of parasites following an anthelmintic treatment; or (iii) natural clearance of the infection, as in these cases it may take 3–7 weeks and 3–9 weeks for negative AG and AB results, respectively. This explains the higher number of dogs testing positive for antibody detection compared to circulating antigen detection [28]. However, as false positive and false negative results may occur in both ELISAs, the positive predictive value is highest when obtaining positive results for both AG and AB detection [28]. This is particularly important in areas with an expected low prevalence, such as in the present case.

**Table 2:** Overview of the serological results for *A. vasorum* tests performed in Europe

Country	Sample size	AB and AG (%)	AB only (%)	AG only (%)	AG or AB (%)	Reference
Romania	1545	0 (0.00)	30 (1.94)	3 (0.19)	33 (2.14)	Present study
Germany	4003	13 (0.32)	77 (1.92)	7 (0.17)	97 (2.42)	[28]
UK	4030	39 (0.97)	90 (2.23)	14 (0.35)	143 (3.55)	[28]
Poland	3345	17 (0.51)	43 (1.29)	26 (0.78)	86 (2.57)	[37]
Italy	712	2 (0.28)	4 (0.56)	0 (0)	6 (0.84)	[24]
Hungary	1247	17 (1.36)	34 (2.73)	22 (1.76)	73 (5.85)	[33]
Switzerland	6136	59 (0.96)	130 (2.12)	74 (1.21)	263 (4.29)	[38]
Slovakia	225	4 (1.10)	7 (3.11)	4 (1.78)	14 (6.22)	[39]
Bulgaria	150	0 (0)	1 (0.67)	0 (0)	1 (0.67)	[40]
Portugal	906	6 (0.66)	12 (1.32)	18 (1.99)	36 (3.97)	[41]
France	2289	26 (1.14)	46 (2.01)	14 (0.61)	86 (3.76)	[42]
Sweden	3885	4 (0.10)	34 (0.88)	20 (0.51)	58 (1.49)	[43]

The dog-related risk factors for *A. vasorum* infection were reviewed on several occasions and exhaustively discussed by Morgan et al. [30]. Generally, age is considered a risk factor, with dogs under 18 months showing a higher risk of infection. Other factors such as the month of diagnosing the infection, or sex were found not to influence the risk of infection [30], as in our study.

Based on the literature, in recent years the number of reports both in wild canids and domestic dogs has increased in Europe. This might represent either a true emergence and/or an increased awareness and surveillance by using recently developed diagnostic procedures. However, such assumptions are not possible for countries where surveillance and/or routine testing were historically absent, as in the case of Romania. Several elements (i.e. climate, definitive and intermediate host density) were incriminated as predictive emergence drivers leading to increased risk for canine *A. vasorum* infections [31]. According to this model, western Romania falls within a low eco-climatic index and the eastern part is within the zero-risk area. However, the authors do not exclude smaller disease foci under favourable microclimatic conditions [31]. It has been previously summarised that the prevalence in dog populations is lower than in fox populations from the same area [32]. Recently, the western part of Romania has been surveyed for the presence of *A. vasorum* in red foxes, *Vulpes vulpes* [26]. The overall prevalence was 4.2%, which was considered at the lower limit compared to other European countries.

Little is known about the presence of *A. vasorum* in the countries surrounding Romania. According to the available epidemiological data, Romania is located at the eastern margin of the geographical distribution of canine angiostrongylosis: on the western side, the presence of *A. vasorum* was confirmed in dogs [33], foxes [34] and golden jackals (*Canis aureus*) [35] from Hungary, while south-eastwards *A. vasorum* was detected in dogs [36] and a golden jackal in Serbia [4].

## Conclusions

The present survey provides a useful update on the epidemiological situation of *A. vasorum* in dogs from Romania. The detection of circulating antigens and/or specific antibodies against *A. vasorum* demonstrates the presence of this parasite in domestic dogs in Romania, in addition to the recent report in foxes [26]. However, so far, no clinical cases have been reported in the country and this may be related to a low awareness among vets. The situation in the eastern half of the country and beyond remains poorly surveyed, and, also based on the absence of dogs seropositive for both antigen and antibody detection, more studies in foxes and domestic dogs are required. Awareness campaigns among vets and owners are essential in order to prevent fatal cases of canine angiostrongylosis.

## Additional files

**Additional file 1: Table S1.** Raw dataset including the information for all dogs included in the study.

**Additional file 2.** Detailed values for all calculated statistical values.

## Data Availability

The datasets used and/or analysed during the present study are available in the additional files associated with this manuscript.
