# Supplementary material for: The first seroepidemiological survey for Angiostrongylus vasorum in domestic dogs from Romania
Source: Parasit Vectors. 2019 May 14;12:224. doi: 10.1186/s13071-019-3481-0 (PMC6515677; doi:10.1186/s13071-019-3481-0)
Supplement: Supplementary file 2 — Additional file 2. Detailed values for all calculated statistical values. [file 13071_2019_3481_MOESM2_ESM.docx]

**Additional file 2**

Comparison of prevalence according to county. Significant results are highlighted in red.

|  | | Χ^2^ | *P* |
| --- | --- | --- | --- |
| All counties  d.f.= 22 | AG | 21.795 | 0.472 |
|  | AB | 34.32 | **0.045** |
|  | AG or AB | 32.999 | 0.061 |
| Positive counties  d.f.= 10 | AG | 12.099 | 0.278 |
|  | AB | 9.018 | 0.530 |
|  | AG or AB | 6.971 | 0.728 |

Comparison of prevalence according to sex.

|  | | Male (n=808) | Female (n=737) |
| --- | --- | --- | --- |
| AG | % | 0 | 0.41 |
|  | 95% CI | - | 0.14-1.19% |
|  | Χ^2^ (d.f.=1) | 1.529 | |
|  | *P* | 0.216 | |
| AB | % | 2.60 | 1.22 |
|  | 95% CI | 1.71-3.94% | 0.64-2.30% |
|  | Χ^2^ (d.f.=1) | 3.153 | |
|  | *P* | 0.075 | |
| AG or AB | % | 2.60 | 1.63 |
|  | 95% CI | 1.71-3.94% | 0.93-2.82% |
|  | Χ^2^ (d.f.=1) | 1.304 | |
|  | *P* | 0.253 | |

Comparison of prevalence according to origin.

|  | | Rural (n=900) | Urban (n=645) |
| --- | --- | --- | --- |
| AG | % | 0.33 | 0 |
|  | 95% CI | 0.11-0.98% | - |
|  | Χ^2^ (d.f.=1) | 0.777 | |
|  | *P* | 0.377 | |
| AB | % | 2.33 | 1.40 |
|  | 95% CI | 1.53-3.54% | 0.74-2.63% |
|  | Χ^2^ (d.f.=1) | 1.278 | |
|  | *P* | 0.258 | |
| AG or AB | % | 2.67 | 1.40 |
|  | 95% CI | 1.80-3.94% | 0.74-2.63% |
|  | Χ^2^ (d.f.=1) | 2.328 | |
|  | *P* | 0.126 | |

Comparison of prevalence according to free access to the environment.

|  | | Free access to environment (n=932) | No free access to environment (n=613) |
| --- | --- | --- | --- |
| AG | % | 0.32 | 0 |
|  | 95% CI | 0.11-0.94% | - |
|  | Χ^2^ (d.f.=1) | 0.664 | |
|  | *P* | 0.414 | |
| AB | % | 1.82 | 2.12 |
|  | 95% CI | 1.14-2.90% | 1.24-3.59% |
|  | Χ^2^ (d.f.=1) | 0.050 | |
|  | *P* | 0.821 | |
| AG or AB | % | 2.15 | 2.12 |
|  | 95% CI | 1.39-3.29% | 1.24-3.59% |
|  | Χ^2^ (d.f.=1) | 0.000 | |
|  | *P* | 1.000 | |

Comparison of prevalence according to housing.

|  | | Outdoor (n=1197) | Indoor (n=340) | Mixed (n=8) |
| --- | --- | --- | --- | --- |
| AG | % | 0.25 | 0 | 0 |
|  | 95% CI | 0.09-0.73% | - | - |
|  | Χ^2^ (d.f.=2) | 0.873 | | |
|  | *P* | 0.646 | | |
| AB | % | 1.67 | 2.94 | 0 |
|  | 95% CI | 1.08-2.57% | 1.61-5.33% | - |
|  | Χ^2^ (d.f.=2) | 2.403 | | |
|  | *P* | 0.300 | | |
| AG or AB | % | 1.92 | 2.94 | 0 |
|  | 95% CI | 1.28-2.87% | 1.61-5.33% | - |
|  | Χ^2^ (d.f.=2) | 1.492 | | |
|  | *P* | 0.474 | | |

Comparison of prevalence according to breed. Significant results are highlighted in red.

|  | | Pure breed (n=296) | Mixed breed (n=17) | Mongrel (n=1232) |
| --- | --- | --- | --- | --- |
| AG | % | 0.34 | 0 | 0.16 |
|  | 95% CI | 0.01-1.87% | - | 0.04-0.59% |
|  | Χ^2^ (d.f.=2) | 0.412 | | |
|  | *P* | 0.813 | | |
| AB | % | 3.72 | 0 | 1.54 |
|  | 95% CI | 1.87-6.55% | - | 0.99-2.40% |
|  | Χ^2^ (d.f.=2) | 6.264 | | |
|  | *P* | **0.043** | | |
| AG or AB | % | 4.05 | 0 | 1.70 |
|  | 95% CI | 2.11-6.97% | - | 1.12-2.59% |
|  | Χ^2^ (d.f.=2) | 6.677 | | |
|  | *P* | **0.035** | | |

Comparison of prevalence according to age. Significant results are highlighted in red.

|  | | | | Puppy (n=16) | | Young (n=251) | | Adult (n=1191) | | Old (n=87) | |
| --- | --- | --- | --- | --- | --- | --- | --- | --- | --- | --- | --- |
| AG | | % | | 0 | | 0 | | 0.25 | | 0 | |
|  |  | 95% CI | | - | | - | | 0.09-0.74% | | - | |
|  |  | Χ^2^ (d.f.=3) | | 0.893 | | | | | | | |
|  |  | *P* | | 0.827 | | | | | | | |
| AB | | % | | 12.50 | | 0.80 | | 2.10 | | 1.15 | |
|  |  | 95% CI | | 1.55-38.35 | | 0.10-2.85% | | 1.43-3.08% | | 0.03-6.24% | |
|  |  | Χ^2^ (d.f.=3) | | 11.537 | | | | | | | |
|  |  | *P* | | **0.009** | | | | | | | |
| AG or AB | | % | | 12.50 | | 0.80 | | 2.35 | | 1.15 | |
|  |  | 95% CI | | 1.55-38.35 | | 0.10-2.85% | | 1.63-3.38% | | 0.03-6.24% | |
|  |  | Χ^2^ (d.f.=3) | | 11.043 | | | | | | | |
|  |  | *P* | | **0.011** | | | | | | | |
|  | | | Shelter (n=750) | | Security (n=432) | | Pet (n=206) | | Shepherd (n=88) | | Hunting (n=69) |
| AG | % | | 0.13 | | 0.46 | | 0 | | 0 | | 0 |
|  | 95% CI | | 0.02-0.75% | | 0.13-1.67% | | - | | - | | - |
|  | Χ^2^ (d.f.=4) | | 2.460 | | | | | | | | |
|  | *P* | | 0.651 | | | | | | | | |
| AB | % | | 1.87 | | 2.31 | | 0.49 | | 5.68 | | 0 |
|  | 95% CI | | 1.12-3.11% | | 1.26-4.21% | | 0.01-2.67% | | 1.87-12.76% | | - |
|  | Χ^2^ (d.f.=4) | | 10.463 | | | | | | | | |
|  | *P* | | **0.033** | | | | | | | | |
| AG or AB | % | | 2.00 | | 2.78 | | 0.49 | | 5.68 | | 0 |
|  | 95% CI | | 1.22-3.27% | | 1.60-4.79% | | 0.01-2.67% | | 1.87-12.76% | | - |
|  | Χ^2^ (d.f.=4) | | 10.401 | | | | | | | | |
|  | *P* | | **0.034** | | | | | | | | |
